# Association between chronic pain and cognitive frailty among middle-aged and elderly individuals: evidence from the China Health and Retirement Longitudinal Study

**DOI:** 10.3389/fnagi.2024.1491120

**Published:** 2024-12-02

**Authors:** Tianjiao Li, Lingxuan Li, Hongyang Xie, Rongyu Ping, Yane Guo, Dongmei Li, Yuwei Zhang, Xiujuan Bai, Bo Sun

**Affiliations:** ^1^Geriatric Neurological Department of the Second Medical Center and National Clinical Research Center for Geriatric Diseases, Chinese PLA General Hospital, Beijing, China; ^2^Beijing Retired Cadre Service Administration, Beijing, China; ^3^Department of Psychology, College of Humanities and Social Sciences, Beijing Forestry University, Beijing, China; ^4^Neurological Department of the Seventh Medical Center, Chinese PLA General Hospital, Beijing, China; ^5^State Key Laboratory of Medical Proteomics Beijing Proteome Research Center, National Center for Protein Sciences (Beijing), Beijing Institute of Lifeomics, Beijing, China; ^6^The 19th Retired Cadre Rehabilitation Center, Beijing, China

**Keywords:** chronic pain, cognitive frailty, middle-aged and elderly population, China Health and Retirement Longitudinal Study, aging

## Abstract

**Background:**

Frailty, particularly cognitive frailty, is an escalating public health issue. Cognitive frailty is defined by the simultaneous presence of physical frailty and cognitive impairment, without a confirmed diagnosis of dementia, and has become a significant geriatric syndrome. This study aimed to explore the association between chronic pain and the risk of cognitive frailty.

**Methods:**

We utilized data from two waves (2011 and 2015) of the China Health and Retirement Longitudinal Study (CHARLS), conducting both cross-sectional and longitudinal analyses involving 17,705 Chinese adults aged 45 years and older. Chronic pain was defined as pain reported at both time points. Cognitive function was evaluated using a questionnaire adapted from the Telephone Interview for Cognitive Status. The frailty index (FI) was derived from a 30-item assessment. Cognitive frailty was characterized by the co-occurrence of cognitive impairment and physical frailty.

**Results:**

Among the 14,285 participants, 5.39% exhibited cognitive frailty at baseline. Both cross-sectional and longitudinal analyses indicated that individuals suffering from chronic pain faced a higher likelihood of developing cognitive frailty compared to those without pain. After adjusting for potential confounders, multivariate models also indicated a higher odds of cognitive frailty for participants with chronic pain.

**Conclusion:**

Chronic pain is significantly associated with an elevated risk of cognitive frailty among middle-aged and elderly individuals. These findings highlight the importance of managing chronic pain to mitigate the risk of cognitive frailty, thereby potentially enhancing the quality of life for the aging population and alleviating the economic burden on families and society.

## 1 Introduction

**Figure 1:**
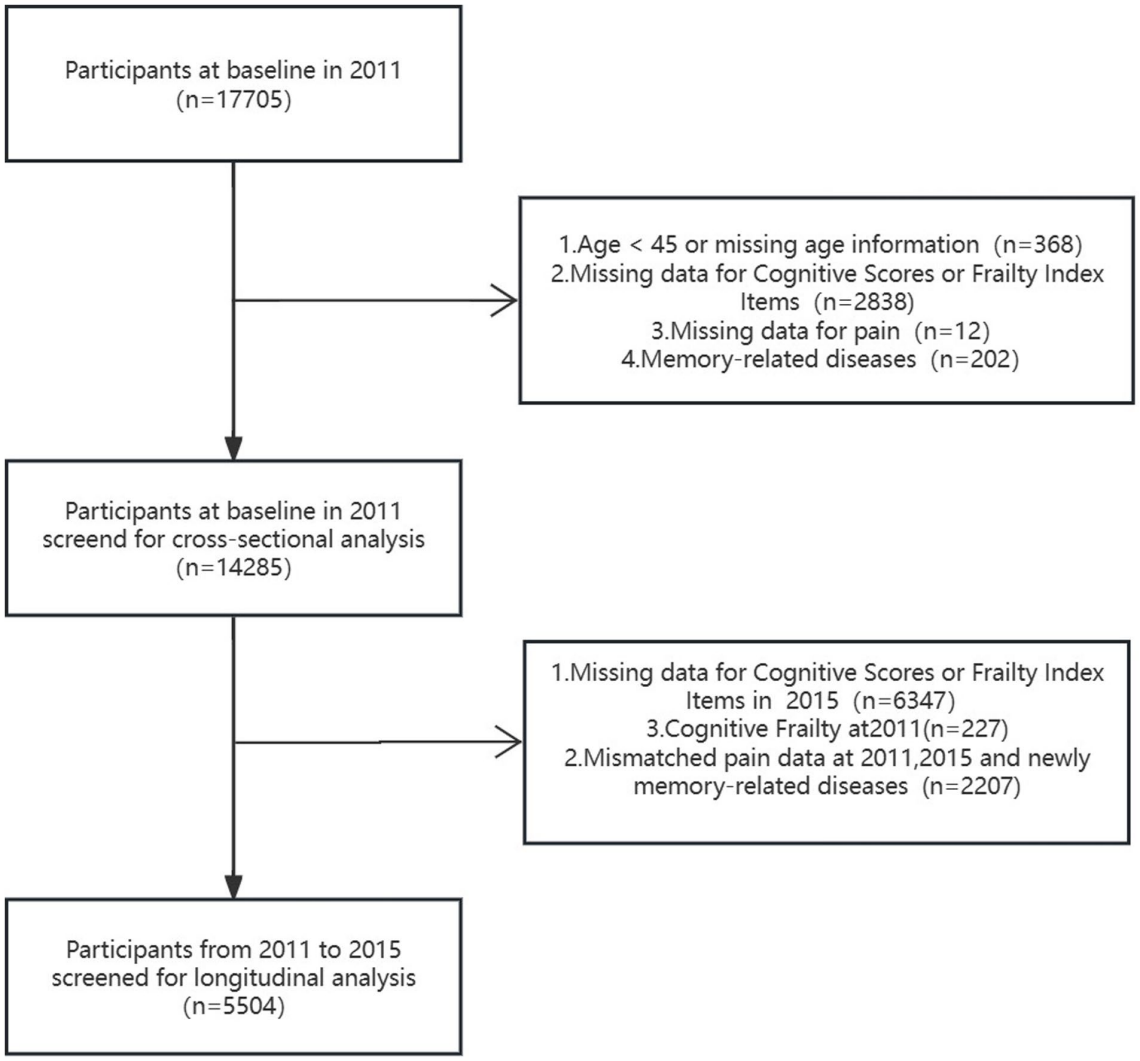
Study flow chart.

With the accelerated population aging, frailty has become a major health problem all over the world. Frailty is defined as a geriatric syndrome characterized by increased susceptibility to stressors due to cumulative decline in multiple physiological systems (Clegg et al., 2013; Robertson et al., 2013). Frailty is a multidimensional geriatric condition that integrates physical, social and cognitive domains (Clegg et al., 2013; Robertson et al., 2013). Cognitive impairment is considered as a component of frailty, and significant association between physical frailty and cognition has been confirmed based on extensive evidence (Panza et al., 2015; Panza et al., 2015; Robertson et al., 2013). The concept of “cognitive frailty” has been proposed by the International Academy on Nutrition and Aging (IANA) and the International Association of Gerontology and Geriatrics (IAGG) in 2013 (Kelaiditi et al., 2013). Cognitive frailty refers to the concurrent presence of both physical frailty and cognitive impairment in the elderly population without definite diagnosis of dementia, which is characterized by reversibility (Kelaiditi et al., 2013). The estimated prevalence of cognitive frailty within the geriatric population in the community is approximately 1.0–12.1%. However, the prevalence significantly increases in clinical settings (Shimada et al., 2016; Feng et al., 2017; Feng et al., 2017; Solfrizzi et al., 2017; Roppolo et al., 2017; Merchant et al., 2017; St John et al., 2017; Montero-Odasso et al., 2016; Delrieu et al., 2016; Fougère et al., 2017). Cognitive frailty is affected by various factors, for example, age, income, marriage status, education level, BMI, comorbidity, disability, self-care ability (Jha et al., 2016; Smith et al., 2015; Li et al., 2018; Kojima et al., 2020; Handayani et al., 2019; Aprahamian et al., 2018; Yang and Chen, 2018; Ge et al., 2020; Jiao et al., 2020; Xiong et al., 2019; Trevisan et al., 2017; Herr et al., 2019; Chiou et al., 2018). Few studies with large sample size illustrated the association between pain and frailty or pain and cognition (Chiou et al., 2018), and until now, there was no research on pain and cognitive frailty (Figure 1).

Pain is an unpleasant sensory and emotional experience. It usually indicates tissue damage, and is prevalent among middle-aged and elderly adults. Chronic pain is a prevalent and significant medical condition worldwide. It is characterized as persistent nociception beyond the expected duration of tissue healing, typically manifesting as pain for a minimum of 3 months (Jackson et al., 2016; Jackson et al., 2015; Vos et al., 2016). Chronic pain is particularly common in elderly adults (≥age 65), and precipitates negative outcomes, including malnutrition, loss of daily functioning, and frailty (Pitcher et al., 2018; Cohen et al., 2021).

As cognitive frailty increases costs and burden on health care systems, it is of vital importance to explore potential factors contributing to cognitive frailty. The association between chronic pain and cognitive frailty remains inconclusive, therefore, prospective cohort studies with large sample size are necessary to investigate the association between chronic pain and cognitive frailty.

In the current study, we used China Health and Retirement Longitudinal Study (CHARLS) and provided both cross-sectional and longitudinal evidence of the association between chronic pain and cognitive frailty.

## 2 Methods

### 2.1 Study population

The CHARLS is designed to gather a comprehensive and high-quality set of microdata pertaining to households and individuals who are 45 years of age or older in China, with the objective of examining the demographic phenomenon of aging within the Chinese population and fostering interdisciplinary research in gerontology. The national baseline survey of CHARLS employed a multi-stage probability to size (PPS) sampling methodology, which was a systematic approach to ensure representativeness in the sample selection. The survey encompassed 450 villages, 150 counties across 28 provinces, and included over 17,000 individuals from approximately 10,000 households. CHARLS is a longitudinal study with biennial to triennial assessments, and to date, it has released data from four distinct survey waves: the initial national baseline survey (Wave 1, 2011), the first follow-up survey (Wave 2, 2013), the second follow-up survey (Wave 3, 2015), and the third follow-up survey (Wave 4, 2018). The CHARLS survey project received ethical approval from the Biomedical Ethics Committee of Peking University (IRB00001052-11015), and all participants were mandated to provide informed consent prior to their involvement in the study.

Extensive details regarding the CHARLS have been documented and published (Hu et al., 2022; Yan et al., 2023). This cohort study utilized two waves of CHARLS data collected in 2011 (wave 1) and 2015 (wave 3). The sample size of Wave 1 was 17,705, from which 3,420 individuals were excluded (including 368 individuals less than 45 years old or with missing age information, 2,838 individuals with missing data for cognitive scores or frailty index items, 12 individuals with missing data for pain and 202 individuals with memory-related diseases). The cross-sectional analysis involved 14,285 participants. For the longitudinal analysis, 8,781 individuals were excluded, including 6,347 individuals with missing data for cognitive scores or frailty index items at wave 3, 227 individuals diagnosed with cognitive frailty at wave 1, and 2,207 individuals with mismatched pain data at wave 1 and 3, or individuals with newly diagnosed memory-related diseases at wave 3.

### 2.2 Assessment of pain

Pain was evaluated utilizing self-reported symptom inventories, querying: Are you often troubled with any body pains (“no” or “yes”)? On what part of your body do you feel pain? Please list all parts of your body where you are currently feeling pain (head, neck, chest, stomach, shoulder, back, waist, buttocks, arm, leg, knees, wrist, fingers, ankle, and toes). We divided the pain status into no pain and baseline pain. Chronic pain was defined as reporting pain both at baseline (wave 1) and follow-up endpoint (wave 3). Pain was evaluated utilizing self-reported symptom inventories, querying: How bad is your pain (if more than one type of pain, ask about the most severe one among them)? The severity of pain is required to be selected from three options of mild, moderate and severe, and patients experiencing pain are requested to respond.

### 2.3 Measurement of cognitive function

Cognitive function was measured at two time points—the CHARLS 2011 baseline survey and the 2015 follow-up survey—using questionnaires adapted from the Telephone Interview for Cognitive Status. The assessment included episodic memory (score range: 0–10 points), orientation (score range: 0–5 points), calculation (score range: 0–5 points), and drawing (score range: 0–1 points). Episodic memory was measured through immediate and delayed recall. The total score range was from 0 to 21 points, with higher scores indicating better cognitive function. All participants were grouped for every 5 years of age. The participants were classified as having mild cognitive impairment (MCI) if their total score fell more than one standard (SD) below age-appropriate norms (Jak et al., 2009); otherwise, they were defined as normal cognition.

### 2.4 Calculation of frailty index

In accordance with previous methods (Searle et al., 2008; Yuan et al., 2023; Donders et al., 2006; Fan et al., 2020), we employed the Frailty Index (FI) to define frailty. We utilized a previously established 30-item frailty index (Yuan et al., 2023), comprising 13 physician-diagnosed health-related deficits, 5 disability indicators, and 12 limitations in activities of daily living (ADLs) and instrumental activities of daily living (IADLs) (Supplementary Table S1). The sum of scores for each indicator was divided by 30 to derive the FI (range: 0–1), with higher scores indicating greater frailty. Thresholds of 0.1 and 0.2 were utilized to interpret identified trajectories, with scores below 0.1 (FI ≤ 0.10) denoting robustness, scores between 0.1 and 0.2 (0.1 < FI ≤ 0.2) signifying pre-frailty, and scores exceeding 0.2 (FI > 0.2) indicating frailty. (Supplementary Table S1).

### 2.5 Cognitive frailty

According to (I.A.N.A/I.A.G.G) international consensus group (Yan et al., 2023). Cognitive frailty was defined as the concurrent presence of both mild cognitive impairment (MCI) and physical frailty (Kelaiditi et al., 2013).

### 2.6 Covariates

According to prior knowledge, we also considered sociodemographic characteristics and health-related factors in our study. Sociodemographic characteristics included age, gender and marital status (married/unmarried; the term “unmarried” includes several marital statuses: “separated,” “unmarried,” “divorced,” and “widowed”). Health-related factors included ever/current smoke, ever/current alcohol, nighttime sleep duration, poor sleep quality and 14 common co-morbidities (cancer, chronic lung diseases, heart disease, stroke, emotional and mental disorders, arthritis, dyslipidemia, hepatic disease, kidney disease, digestive system disease, asthma, memory-related disease, hypertension, and hyperglycemia). Poor sleep quality was assessed according to the response “my sleep was restless,” and divided into four groups according to the amount of time. Total nighttime sleep duration data were obtained from the question “During the past month, how many hours of actual sleep did you get at night (average hours for one night)?” Body mass index (BMI) was defined as the weight divided by the square of height (kg/m^2^). Depression was evaluated by the 10-item Center for Epidemiologic Studies Depression Scale (CESD-10), with a total score of 30. Health insurance status was ascertained via the Health Insurance Medical Insurance Program survey, wherein participants were required to identify their insurance coverage from a predefined list of options. The classification included: (1) Urban employee medical insurance (yi-bao); (2) Urban resident medical insurance; (3) New cooperative medical insurance (he-zuo-yi-liao); (4) Urban and rural resident medical insurance; (5) Government medical insurance (gong-fei); (6) Medical aid; (7) Private medical insurance procured by the work unit; (8) Private medical insurance procured by the individual; (9) Urban non-employed persons’ health insurance; (10) Other specified medical insurance; and (11) No insurance. Participants who indicated “No insurance” were coded as “No,” whereas all other responses were coded as “Yes” for the presence of insurance coverage.

### 2.7 Statistical analysis

Quantitative data with a normal distribution were described using the mean and standard deviation (SD), while non-normally distributed data were presented using the median (interquartile range). Qualitative data were reported as percentages. Group comparisons between the chronic pain and no-pain groups were carried out using one-way analysis of variance and chi-square tests.

In cross-sectional study, a logistic regression model was employed to investigate the association between chronic pain and cognitive frailty (Wave 1, 2011), and expressed as odds ratios (OR) and 95% confidence intervals (CI). Longitudinal data from 2011 and 2015 were analyzed using logistic regression models to explore the relationship between chronic pain and cognitive frailty. Four different models with various combinations of covariates were utilized. Specifically, Model 1 included only chronic pain; Model 2 included age, gender and marital status; Model 3 further included BMI, waist, education, smoking and alcohol history and insurance; Model 4 further included nighttime sleep duration, poor sleep quality, life-satisfy and CESD score.

We employed logistic regression analysis (model 4) to perform subgroup analyses on the baseline (2011) and follow-up (2015) datasets. The baseline analysis incorporated gender, age (with a cutoff of 60 years old), marital status, and BMI (with a cutoff of 30 kg/m^2^) as categorical variables. The sample for the subgroup logistic regression analysis is consistent with the sample used in the overall logistic regression. In the subgroup analysis conducted in 2015, the baseline frailty and mild cognitive impairment (MCI) subgroup did not include subjects who were diagnosed with cognitive frailty at baseline, as these individuals were subsequently excluded during the follow-up period. The 2015 dataset analysis included these variables along with the addition of baseline frailty and mild cognitive impairment (MCI) as covariates. The aim was to evaluate the influence of these variables on the chronic pain-cognitive frailty association within defined subgroups.

All statistical analyses were conducted using R software (version 4.4.0; R Foundation for Statistical Computing)^1^ and Free Statistics software (version2.01; Beijing Free Clinical Medical Technology Co., Ltd.), with a significance level set at 0.05 for all tests.

## 3 Results

Table 1 presented the characteristics of the 14,285 participants, categorized into non-pain (9,745) and pain groups (4,540), and further stratified into mild (1,166), moderate (1,651), and severe (1,723) pain subgroups. Significant differences were observed across groups in gender, age, marital status, education, alcohol and smoking habits, insurance coverage, sleep quality, life satisfaction, CESD, nighttime sleep duration, cognitive score, frailty index (FI), frailty, MCI, and cognitive frailty (*p* < 0.05). However, there were no significant differences in BMI and waist circumference among the groups (*p* > 0.05). Individuals with cognitive frailty accounted for 5.39% (770) at baseline (wave 1).

**Table 1:** Baseline characteristics of study population in 2011(wave 1).

Variables	Non-pain (*n* = 9,745)	Pain (*n* = 4,540)			*p*-value
		Mild (*n* = 1,166)	Moderate (*n* = 1,651)	Severe (*n* = 1,723)	
Gender, n (%)					< 0.001
Female	4,624 (47.5)	682 (58.5)	1,032 (62.5)	1,072 (62.3)	
Male	5,113 (52.5)	484 (41.5)	618 (37.5)	650 (37.7)	
Age, M ± SD	59.1 ± 9.8	59.2 ± 9.3	59.6 ± 9.3	60.3 ± 9.6	< 0.001
Marital, n (%)					< 0.001
Married	8,604 (88.3)	1,010 (86.6)	1,411 (85.5)	1,478 (85.8)	
Unmarried	1,141 (11.7)	156 (13.4)	240 (14.5)	245 (14.2)	
Education, n (%)					< 0.001
Illiterate	2,307 (23.7)	339 (29.1)	526 (31.9)	647 (37.6)	
Primary school	3,667 (37.6)	526 (45.1)	706 (42.8)	751 (43.6)	
Middle/high school	3,437 (35.3)	288 (24.7)	400 (24.2)	315 (18.3)	
Junior college or above	329 (3.4)	13 (1.1)	19 (1.2)	10 (0.6)	
Ever/current drink, n (%)					< 0.001
No	6,330 (65)	793 (68)	1,198 (72.6)	1,258 (73)	
Yes	3,415 (35)	373 (32)	453 (27.4)	465 (27)	
Ever/current smoke, n (%)					< 0.001
No	5,692 (58.4)	727 (62.3)	1,095 (66.3)	1,111 (64.5)	
Yes	4,052 (41.6)	439 (37.7)	556 (33.7)	612 (35.5)	
Insurance, n (%)					0.016
No	589 (6)	98 (8.4)	112 (6.8)	110 (6.4)	
Yes	9,156 (94)	1,068 (91.6)	1,539 (93.2)	1,613 (93.6)	
Poor sleep quality, n (%)					< 0.001
Most or all of the time	1,337 (13.7)	297 (25.5)	492 (29.9)	628 (36.7)	
Occasionally or a moderate amount of the time	1,215 (12.5)	212 (18.2)	361 (21.9)	348 (20.3)	
Rarely or none of the time	5,562 (57.2)	455 (39.1)	500 (30.3)	490 (28.6)	
Some or a little of the time	1,617 (16.6)	199 (17.1)	295 (17.9)	247 (14.4)	
Life-satisfy, n (%)					< 0.001
Poor	1,038 (10.7)	112 (9.6)	224 (13.6)	327 (19)	
Fair	6,568 (67.4)	821 (70.4)	1,198 (72.6)	1,127 (65.4)	
Good	2,139 (21.9)	233 (20)	229 (13.9)	269 (15.6)	
CESD, M ± SD	6.6 ± 5.3	10.1 ± 6.2	12.1 ± 6.5	13.8 ± 6.8	< 0.001
BMI, M ± SD	23.5 ± 3.8	23.3 ± 3.8	23.4 ± 3.8	23.3 ± 3.9	0.172
Waist circumference, M ± SD	84.6 ± 12.3	84.3 ± 12.9	83.8 ± 12.8	83.9 ± 12.9	0.065
Nighttime sleep duration, M ± SD	6.6 ± 1.7	6.2 ± 2.0	5.9 ± 2.0	5.7 ± 2.2	< 0.001
cognition, M ± SD	10.9 ± 4.3	9.9 ± 4.2	9.6 ± 4.2	8.6 ± 4.3	< 0.001
Frailty, n (%)					< 0.001
No	8,806 (90.4)	897 (76.9)	1,047 (63.4)	904 (52.5)	
Yes	939 (9.6)	269 (23.1)	604 (36.6)	819 (47.5)	
MCI, n (%)					< 0.001
No	8,322 (85.4)	946 (81.1)	1,302 (78.9)	1,202 (69.8)	
Yes	1,423 (14.6)	220 (18.9)	349 (21.1)	521 (30.2)	
Frailty + MCI, n (%)					< 0.001
No	9,521 (97.7)	1,091 (93.6)	1,482 (89.8)	1,421 (82.5)	
Yes	224 (2.3)	75 (6.4)	169 (10.2)	302 (17.5)	
FI, median (IQR)	2.0 (1.0, 4.0)	4.0 (2.0, 6.0)	5.0 (3.0, 8.0)	6.0 (4.0, 9.0)	< 0.001

BMI, body mass index; CESD, Center for Epidemiologic Studies Depression; M ± SD, mean ± standard deviation; MCI, Mild Cognitive Impairment; FI, Frailty Index.

Table 2 showed the relationship between pain and cognitive frailty at the baseline period (2011). In the unadjusted model (Model 1), individuals with mild, moderate, and severe pain demonstrated significantly higher odds of cognitive frailty compared to those without pain, with corresponding odds ratios (ORs) of 2.92 (95% CI 2.23–3.82), 4.85 (95% CI 3.94–5.96), and 9.03 (95% CI 7.53–10.83) (all with *p* < 0.001). Even after comprehensive adjustments for age, gender, marital status, BMI, waist circumference, education, smoking and drinking habits, insurance status, nighttime sleep duration, poor sleep quality, life satisfaction, and CESD score (Model 4), the ORs for mild, moderate, and severe pain remained significant at 2.53 (95% CI 1.81–3.53), 2.78 (95% CI 2.1–3.68), and 4.68 (95% CI 3.63–6.03) (all with *p* < 0.001).

**Table 2:** Logistic regression model on pain and Cognitive Frailty at 2011.

Pain	Model							
	Model1		Model2		Model3		Model4	
	OR (95% CI)	*p*-value	OR(95% CI)	*P*-value	OR(95% CI)	*P*-value	OR(95% CI)	*P*-value
Non-pain	1(Ref)		1(Ref)		1(Ref)		1(Ref)	
Mild	2.92 (2.23 ~ 3.82)	<0.001	2.69 (2.05 ~ 3.53)	<0.001	2.94 (2.17 ~ 3.97)	<0.001	2.53 (1.81 ~ 3.53)	<0.001
Moderate	4.85 (3.94 ~ 5.96)	<0.001	4.24 (3.44 ~ 5.24)	<0.001	4.16 (3.25 ~ 5.33)	<0.001	2.78 (2.1 ~ 3.68)	<0.001
Severe	9.03 (7.53 ~ 10.83)	<0.001	8.03 (6.67 ~ 9.67)	<0.001	8.08 (6.51 ~ 10.02)	<0.001	4.68 (3.63 ~ 6.03)	<0.001

Model 1: No adjustment; Model 2: Adjusted for age, gender, and marital status; Model 3: Model 2 + BMI, waist, education, ever/current smoke, ever/current alcohol, insurance; Model 4: Model 3 + Nighttime sleep duration, poor sleep quality, life-satisfy and CESD score.

**Figure 2:**
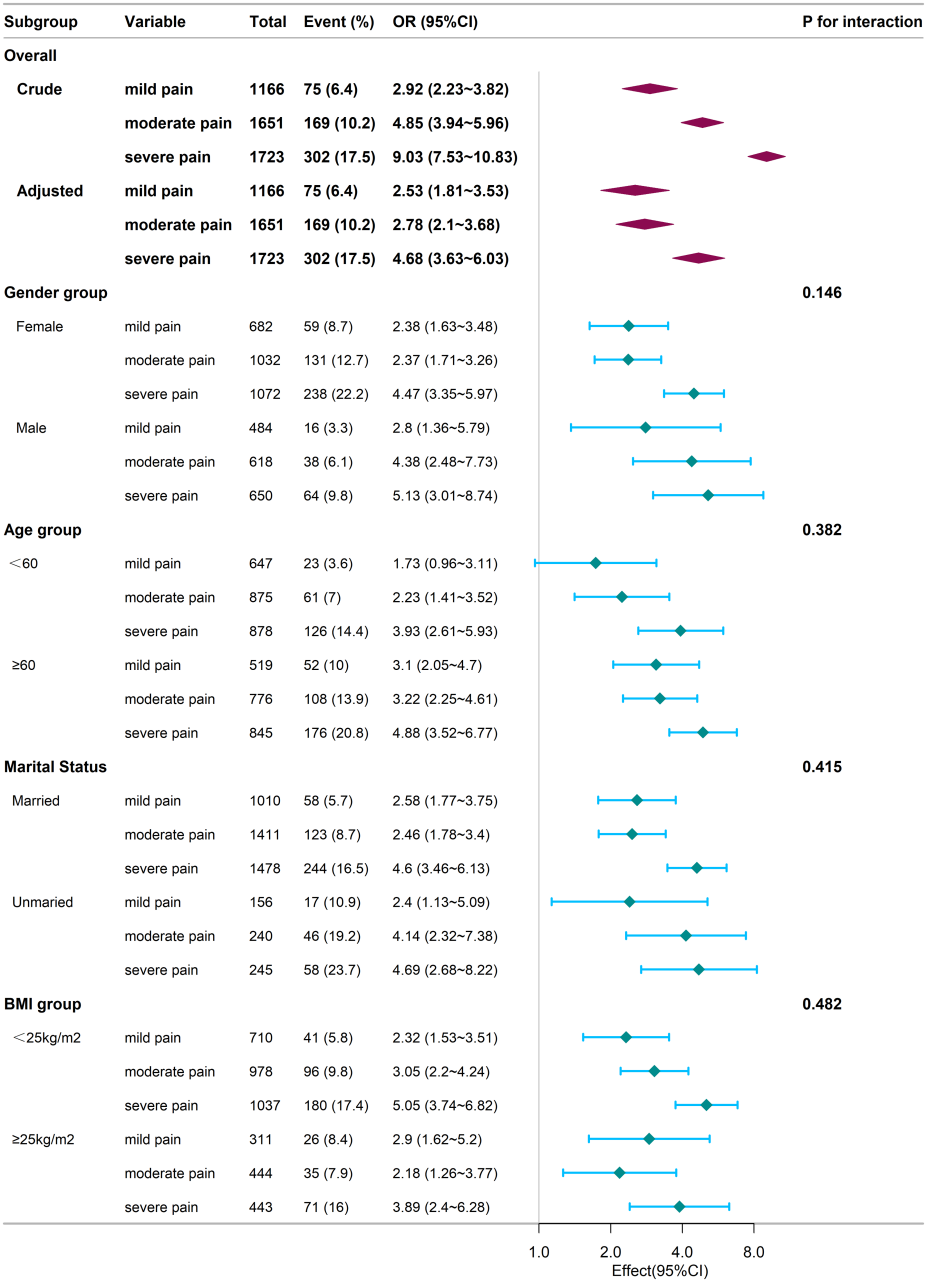
Subgroup analysis of the relationship between chronic pain and cognitive frailty at baseline. Adjustments for confounders were conducted using Model 4. The logistic regression analysis for subgrouping was performed using an identical sample as that employed in the baseline-wide logistic regression. Square symbols are employed to represent the odds ratios (ORs), while horizontal lines delineate the 95% confidence intervals (CIs). Diamond shapes are used to depict the population-level odds ratios, with the points at the vertices of the diamonds indicating the 95% confidence intervals. CI, Confidence Interval; OR, Odds Ratio; BMI, Body Mass Index.

Figure 2 presents additional insights from subgroup analysis. For the subgroup aged <60, no statistically significant difference was found between the mild pain group and the no pain group (*p* > 0.05). In contrast, within the other subgroups, both the mild, moderate, and severe pain groups demonstrated significantly higher odds of cognitive frailty compared to individuals without pain (*p* < 0.05).

Table 3 expanded the analysis to the 2015 follow-up period, providing further insight into the association between pain and cognitive frailty. In the unadjusted model (Model 1), individuals experiencing mild, moderate, and severe pain demonstrated significantly elevated odds of cognitive frailty compared to those without pain, with ORs of 5.87 (95% CI 3.51–9.80), 6.10 (95% CI 4.02–9.25), and 10.63 (95% CI 7.42–15.21), respectively (all with *p* < 0.001). After comprehensive adjustments (Model 4), individuals with mild, moderate, and severe pain still exhibited significantly higher odds of cognitive frailty compared to those without pain, with ORs of 3.15 (95% CI 1.68–5.92), 2.03 (95% CI 1.14–3.62), and 5.14 (95% CI 3.12–8.48), respectively (all with *p* < 0.05).

**Table 3:** Logistic regression model on pain and Cognitive Frailty at 2015.

Pain	Model							
	Model1		Model2		Model3		Model4	
	OR (95% CI)	*P*-value	OR(95% CI)	*P*-value	OR(95% CI)	*P*-value	OR(95% CI)	*P*-value
Non-pain	1(Ref)		1(Ref)		1(Ref)		1(Ref)	
Mild	5.87 (3.51 ~ 9.8)	<0.001	5.03 (2.98 ~ 8.5)	<0.001	4.57 (2.57 ~ 8.14)	<0.001	3.15 (1.68 ~ 5.92)	<0.001
Moderate	6.1 (4.02 ~ 9.25)	<0.001	4.29 (2.78 ~ 6.61)	<0.001	3.87 (2.39 ~ 6.26)	<0.001	2.03 (1.14 ~ 3.62)	0.016
Severe	10.63 (7.42 ~ 15.21)	<0.001	8.97 (6.19 ~ 13)	<0.001	8.9 (5.84 ~ 13.55)	<0.001	5.14 (3.12 ~ 8.48)	<0.001

Model 1: No adjustment; Model 2: Adjusted for age, gender, and marital status; Model 3: Model 2 + BMI, waist, education, ever/current smoke, ever/current alcohol, insurance; Model 4: Model 3 + Nighttime sleep duration, poor sleep quality, life-satisfy and CESD score.

**Figure 3:**
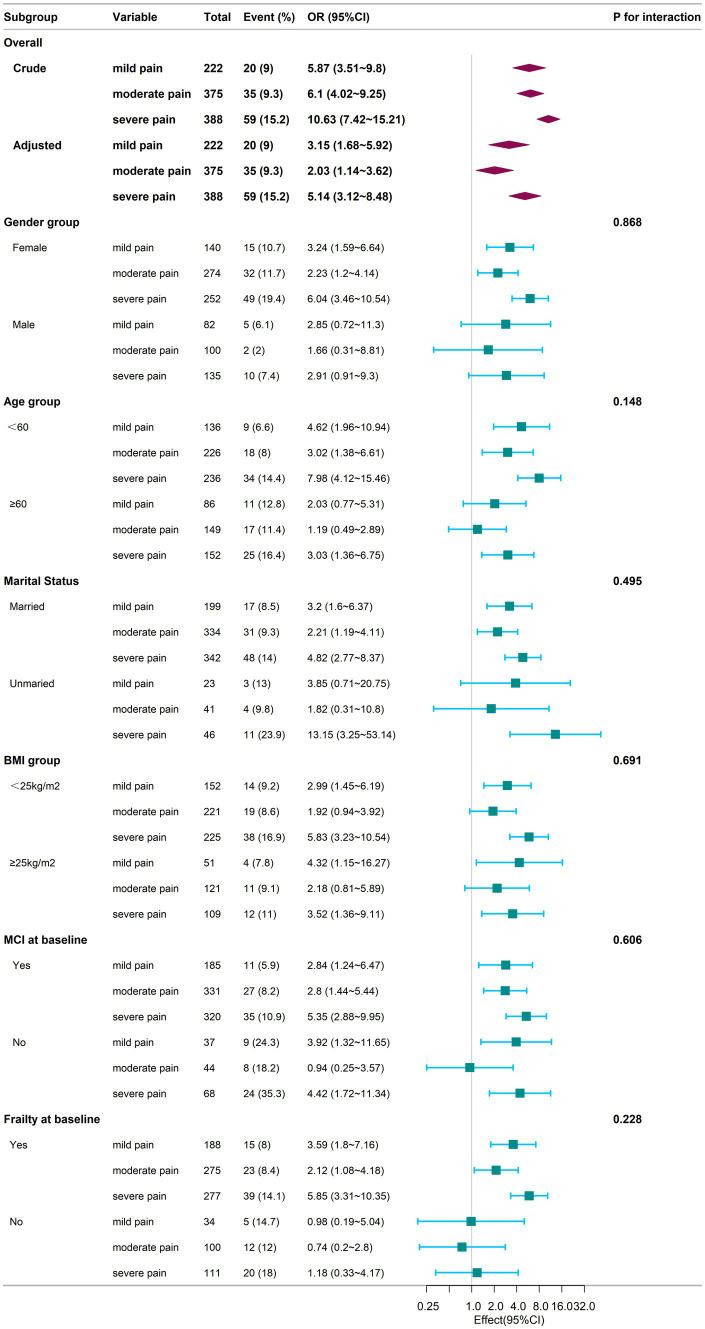
Subgroup analysis of the association between chronic pain and cognitive frailty during the follow-up period. Adjustments for confounding variables were performed using Model 4. The sample for the subgroup logistic regression analysis mirrors that of the overall logistic regression conducted during the follow-up phase. The baseline frailty and mild cognitive impairment (MCI) subgroup excludes individuals diagnosed with cognitive frailty at baseline, as these subjects were not included during the follow-up due to subsequent exclusion criteria. Square symbols represent the odds ratios (ORs), and horizontal lines indicate the 95% confidence intervals (CIs). Diamond shapes depict the population-level odds ratios, with the points along the edges of the diamonds representing the 95% confidence intervals. CI, Confidence Interval; OR, Odds Ratio; BMI, Body Mass Index; MCI, Mild Cognitive Impairment.

Figure 3 provided additional insights from subgroup analysis. In the subgroup of individuals aged <60 years, married individuals, those with baseline mild cognitive impairment (MCI) and those with baseline frailty, the odds of experiencing cognitive frailty were significantly higher for individuals with mild, moderate, and severe pain compared to those without pain (*p* < 0.05). Conversely, in the subgroup of individuals aged ≥60 years, individuals with moderate and severe pain demonstrated significantly elevated odds of cognitive frailty compared to those without pain (*p* < 0.05), while no significant difference was found in the odds of cognitive frailty between those with mild pain and those without pain (*p >* 0.05). In the male and frailty at baseline subgroups, individuals experiencing mild, moderate, and severe pain did not display significantly increased odds of cognitive frailty compared to those without pain (*p* > 0.05). However, in the unmarried subgroup, individuals experiencing severe pain showed significantly elevated odds of cognitive frailty compared to those without pain (*p* < 0.05), while no significant difference was observed for individuals with mild and moderate pain compared to those without pain (*p* > 0.05). Furthermore, in the subgroups based on BMI categorized as ≥25 kg/m^2^ and < 25 kg/m^2^, individuals experiencing mild and severe pain exhibited significantly higher odds of cognitive frailty compared to those without pain (*p* < 0.05). Conversely, individuals experiencing moderate pain did not demonstrate significantly increased odds of cognitive frailty compared to those without pain (*p* > 0.05). There were no significant interactions between the groups (*p* > 0.05).

## 4 Discussion

According to our cross-sectional study, the incidence of cognitive frailty was significantly higher in individuals with mild, moderate, and severe pain than those without pain, and in our longitudinal study, individuals with mild, moderate, and severe pain had a higher risk of developing cognitive frailty compared to those without pain. Subgroup analysis revealed that age, gender, marital status, BMI, baseline MCI and baseline frailty might be promoting/preventing factors to develop cognitive frailty.

“Cognitive frailty” was proposed by the IANA and the IAGG in 2013, which clarified its 2 criteria, namely: (1) co-existing of physical frailty and cognitive impairment; (2) exclusion of concurrent dementia (Kelaiditi et al., 2013). Thus, the simultaneous presence of both physical frailty and cognitive impairment with the feature of reversibility were required to diagnose cognitive frailty (Kelaiditi et al., 2013). In the community setting, the incidence of cognitive frailty in the elderly was approximately 1.0–12.1% (Shimada et al., 2016; Feng et al., 2017; Feng et al., 2017; Solfrizzi et al., 2017; Montero-Odasso et al., 2016; Delrieu et al., 2016; Fougère et al., 2017). However, in clinical settings, the prevalence of cognitive frailty could be as high as 10.7–39.7% (Roppolo et al., 2017; Merchant et al., 2017; St John et al., 2017; Jha et al., 2016). In the present study, the prevalence of cognitive frailty was 5.39%, which was in accordance with previous studies. Until now, there were no studies of cognitive frailty with large-sample size in China, and this study filled the domestic gap.

Few studies investigated the association between pain and cognitive frailty. Previous work revealed a significant positive correlation between chronic pain and frailty (Blyth et al., 2008; Coelho et al., 2017; Wade et al., 2017). Empirical studies disclosed a robust association between the prevalence of chronic pain and the incidence of frailty, with an estimated 40–50% of the frail elderly population concurrently enduring chronic pain (Chang et al., 2011; Shega et al., 2013). Elderly individuals with chronic pain were at higher risk to developing frailty compared to those without chronic pain. In a longitudinal cohort study with 8-year follow-up, pain alone was reported to cause a fairly high percentage of frailty compared to the individuals without pain (Wade et al., 2017). A study based on Health and Retirement Study (HRS) revealed that persistent severe pain trajectory was associated with poorer overall cognition, memory and calculation ability (Sun et al., 2024). Another longitudinal study confirmed that older adults exposed to severe long-term pain had a significantly faster cognitive decline (Rong et al., 2021). A cross-sectional study investigated the association between pain, cognition and frailty, and discovered that the association between pain and cognitive function became non-significant after adjustment for age, sex and education level (Smith et al., 2015). However, the above work was cross-sectional and with relatively small sample size. A variety of studies confirmed positive correlation between chronic pain and frailty, and positive correlation between chronic pain and cognitive impairment. Therefore, we hypothesized positive correlation between chronic pain and cognitive frailty, and eventually revealed that the incidence of cognitive frailty was significantly higher in individuals with chronic pain, and individuals with chronic pain had a higher risk of developing cognitive frailty.

The mechanism correlating pain and frailty/cognition also supported the association between chronic pain and cognitive frailty. First, persistent pain caused impaired mobility, decreased resting metabolic rate, and decreased intake. Second, chronic pain activated the hypothalamic pituitary adrenal (HPA) axis, which increased cortisol levels (McBeth et al., 2005; Choi et al., 2010; Varadhan et al., 2008). HPA axis dysregulation was also a keypoint in the onset of frailty (McBeth et al., 2005; Choi et al., 2010; Varadhan et al., 2008). Thirdly, persistent pain and frailty were linked through the immune-inflammatory response (Edwards et al., 2008). The biological mechanisms of cognitive impairment are also complicated. For example, noradrenaline (NA), produced by the locus coeruleus (LC), mediates cognitive reserve. Progressive degeneration of the LC with resulting NA deficiency leads to gradual cognitive impairment (López-Ortiz et al., 2024). Furthermore, the ventral tegmental area (VTA) which contains dopaminergic (DA) neurons is disrupted in AD. The disconnection between VTA and other brain regions leads also to cognitive decline (López-Ortiz et al., 2024). Additionally, chronic pain could induce neuropathological modifications, including alterations in gray matter volume and the integrity of the anterior white matter tracts. Such alterations might disrupt the operational efficacy of neural networks integral to cognitive processes (Malfliet et al., 2017; Gomez-Beldarrain et al., 2016). Furthermore, these neuroanatomical changes also impacted cognitive reserve, potentially precipitating a deterioration in cognitive function (Gomez-Beldarrain et al., 2016).

Pain-depression and depression-frailty relationships have been widely investigated (Landi et al., 2005; Herrick et al., 2004). Researchers found higher risk of depressive symptoms in frail older adults with pain (Malfliet et al., 2017). In the present study, we adjusted the item of depression and reached the same results.

The current investigation boasted several methodological merits. Initially, the research harnessed data from the CHARLS, a dataset that was emblematic of the national demographic, thereby enabling a robust examination of the nexus between chronic pain and cognitive frailty among the Chinese geriatric populace. Secondly, the study’s sample size eclipsed that of numerous comparable studies, bolstering the statistical robustness of the findings. Thirdly, the methodology included both cross-sectional and longitudinal analyses, which collectively enhanced the persuasiveness of the evidence linking chronic pain to cognitive frailty.

Conversely, the study was not devoid of limitations. The retrospective nature of the research precluded the comprehensive adjustment for all potential confounding factors. Moreover, a considerable proportion of the CHARLS data exhibited gaps or incompleteness, potentially skewing the results. Furthermore, the diagnoses within the CHARLS database predominantly rely on questionnaires and individual self-reports from subjects, which inevitably introduces recall bias and misclassifies some untreated patients as part of the healthy population, thereby exerting a partial influence on the outcomes. Additionally, the study did not delineate the specific anatomical sites of pain nor did it track the progression of pain over the four-year observation period. Future prospective cohort studies are warranted to more intricately dissect the interplay between distinct chronic pain sites or the trajectory of chronic pain and its impact on cognitive frailty.

## 5 Conclusion

In the present study, we discovered that the incidence of cognitive frailty was significantly higher in people suffered from chronic pain, and people with chronic pain had a higher risk of developing cognitive frailty in middle-aged and older Chinese population. As chronic pain may increase the risk of cognitive frailty, management of chronic pain is of vital importance in preventing cognitive frailty, improving the quality of life in middle-aged and older population and reducing economic burden on families and society.

## Data Availability

The raw data supporting the conclusions of this article will be made available by the authors, without undue reservation.

## Footnotes

^1^ http://www.R-­project.org

## References

[ref1] Aprahamian I. Sassaki E. dos Santos M. F. Izbicki R. Pulgrossi R. C. Biella M. M.. (2018). Hypertension and frailty in older adults. J. Clin. Hypertens. 20, 186–192. doi: 10.1111/jch.13135, PMID: 29105991 PMC8031203

[ref2] Blyth F. M. Rochat S. Cumming R. G. Creasey H. Handelsman D. J. Le Couteur D. G.. (2008). Pain, frailty and comorbidity in older men: the CHAMP study. Pain 140, 224–230. doi: 10.1016/j.pain.2008.08.011, PMID: 18835100

[ref3] Chang C. I. Chan D. C. Kuo K. N. Hsiung C. A. Chen C. Y. (2011). Prevalence and correlates of geriatric frailty in a northern Taiwan community. J. Formos. Med. Assoc. 110, 247–257. doi: 10.1016/S0929-6646(11)60037-5 21540007

[ref4] Chiou J. H. Liu L. K. Lee W. J. Peng L. N. Chen L. K. (2018). What factors mediate the interrelationship between frailty and pain in cognitively and functionally sound older adults? A prospective longitudinal ageing cohort study in Taiwan. BMJ Open 8:e018716. doi: 10.1136/bmjopen-2017-018716, PMID: 29453297 PMC5829604

[ref5] Choi C.-J. Knudsen R. Oda K. Fraser G. E. Knutsen S. F. (2010). The association between incident self-reported fibromyalgia and nonpsychiatric factors: 25 years follow-up of the Adventist health study. J. Pain 11, 1282–1290. doi: 10.1016/j.jpain.2010.03.002 20400378 PMC2946422

[ref6] Clegg A. Young J. Iliffe S. Rikkert M. O. Rockwood K. (2013). Frailty in elderly people. Lancet 381, 752–762. doi: 10.1016/S0140-6736(12)62167-9, PMID: 23395245 PMC4098658

[ref7] Coelho T. Paul C. Gobbens R. J. J. Fernandes L. (2017). Multidimensional frailty and pain in community-dwelling elderly. Pain Med. 18, 693–701. doi: 10.1111/pme.12746, PMID: 25800906

[ref8] Cohen S. P. Vase L. Hooten W. M. (2021). Chronic pain: an update on burden, best practices, and new advances. Lancet 397, 2082–2097. doi: 10.1016/S0140-6736(21)00393-7, PMID: 34062143

[ref9] Delrieu J. Andrieu S. Pahor M. Cantet C. Cesari M. Ousset P. J.. (2016). Neuropsychological profile of "cognitive frailty" subjects in MAPT study. J. Prev Alzheimers Dis. 3, 151–159. doi: 10.14283/jpad.2016.94, PMID: 27547746 PMC4991881

[ref10] Donders A. R. van der Heijden G. J. Stijnen T. Moons K. G. (2006). Review: a gentle introduction to imputation of missing values. J. Clin. Epidemiol. 59, 1087–1091. doi: 10.1016/j.jclinepi.2006.01.014, PMID: 16980149

[ref11] Edwards R. R. Kronflfli T. Haythornthwaite J. A. Smith M. T. McGuire L. Page G. G. (2008). Association of catastrophizing with interleukin-6 responses to acute pain. Pain 140, 135–144. doi: 10.1016/j.pain.2008.07.024, PMID: 18778895 PMC2659503

[ref12] Fan J. Yu C. Guo Y. Bian Z. Sun Z. Yang L.. (2020). Frailty index and all-cause and cause-specific mortality in Chinese adults: a prospective cohort study. Lancet Public Health 5, e650–e660. doi: 10.1016/S2468-2667(20)30113-4, PMID: 33271078 PMC7708389

[ref13] Feng L. Nyunt M. S. Gao Q. Feng L. Lee T. S. Tsoi T.. (2017). Physical frailty, cognitive impairment, and the risk of neurocognitive disorder in the Singapore longitudinal ageing studies. J. Gerontol. A Biol. Sci. Med. Sci. 72, S95–S101. doi: 10.1093/gerona/glx005, PMID: 27013397

[ref14] Feng L. Zin Nyunt M. S. Gao Q. Feng L. Yap K. B. Ng T. P. (2017). Cognitive frailty and adverse health outcomes: findings from the Singapore longitudinal ageing studies (SLAS). J. Am. Med. Dir. Assoc. 18, 252–258. doi: 10.1016/j.jamda.2016.09.015 27838339

[ref15] Fougère B. Daumas M. Lilamand M. Sourdet S. Delrieu J. Vellas B.. (2017). Association between frailty and cognitive impairment: cross-sectional data from Toulouse frailty day hospital. J. Am. Med. Dir. Assoc. 18, 990.e1–990.e5. doi: 10.1016/j.jamda.2017.06.024 28797589

[ref16] GeM.ZhangY.ZhaoW.YueJ.HouL.XiaX.. (2020). Prevalence and its associated factors of physical frailty and cognitive impairment: findings from the West China health and aging trend study (WCHAT). J. Nutr. Health Aging 24, 525–533. doi: 10.1007/s12603-020-1363-y, PMID: 32346692

[ref17] Gomez-Beldarrain M. Oroz I. Zapirain B. G. Ruanova B. F. Fernandez Y. G. Cabrera A.. (2016). Right fronto-insular white matter tracts link cognitive reserve and pain in migraine patients. J. Headache Pain 17:4. doi: 10.1186/s10194-016-0593-1, PMID: 26830863 PMC4735096

[ref18] Handayani S. Harun Y. Mukhlisa M. Bahar E. (2019). Factors that influence cognitive function in epilepsy patients at neurology clinic Mohammad Hoesin hospital Palembang. J. Neurol. Sci. 405:116409. doi: 10.1016/j.jns.2019.10.903

[ref19] Herr M. Cesari M. Landre B. Ankri J. Vellas B. Andrieu S.. (2019). Factors associated with changes of the frailty status after age 70: findings in the MAPT study. Ann. Epidemiol. 34, 65–70.e1. doi: 10.1016/j.annepidem.2019.03.008, PMID: 31005551

[ref20] Herrick C. Steger-May K. Sinacore D. R. Brown M. Schechtman K. B. Binder E. F. (2004). Persistent pain in frail older adults after hip fracture repair. J. Am. Geriatr. Soc. 52, 2062–2068. doi: 10.1111/j.1532-5415.2004.52566.x, PMID: 15571543

[ref21] Hu Y. Peng W. Ren R. Wang Y. Wang G. (2022). Sarcopenia and mild cognitive impairment among elderly adults: The first longitudinal evidence from CHARLS. J. Cachexia. Sarcopenia Muscle 13, 2944–2952. doi: 10.1002/jcsm.13081, PMID: 36058563 PMC9745544

[ref22] Jackson T. Thomas S. Stabile V. Han X. Shotwell M. McQueen K. (2015). Prevalence of chronic pain in low-income and middle-income countries: a systematic review and meta-analysis. Lancet 385:S10. doi: 10.1016/S0140-6736(15)60805-4, PMID: 26313056

[ref23] Jackson T. Thomas S. Stabile V. Shotwell M. Han X. McQueen K. (2016). A systematic review and Meta-analysis of the global burden of chronic pain without clear etiology in low-and middle-income countries: trends in heterogeneous data and a proposal for new assessment methods. Anesth. Analg. 123, 739–748. doi: 10.1213/ANE.0000000000001389, PMID: 27537761

[ref24] Jak A. J. Bondi M. W. Delano-Wood L. Wierenga C. Corey-Bloom J. Salmon D. P.. (2009). Quantification of five neuropsychological approaches to defining mild cognitive impairment. Am. J. Geriatr. Psychiatry 17, 368–375. doi: 10.1097/JGP.0b013e31819431d5, PMID: 19390294 PMC2743175

[ref25] Jha S. R. Hannu M. K. Gore K. Chang S. Newton P. Wilhelm K.. (2016). Cognitive impairment improves the predictive validity of physical frailty for mortality in patients with advanced heart failure referred for heart transplantation. J. Heart Lung Transplant. 35, 1092–1100. doi: 10.1016/j.healun.2016.04.008, PMID: 27282417

[ref26] Jiao J. Wang Y. Zhu C. Fangfang L. Minglei Z. Xianxiu W.. (2020). Prevalence and associated factors for frailty among elder patients in China: a multicentre cross-sectional study. BMC Geriatr. 20:10. doi: 10.21203/rs.2.13603/v3 32164595 PMC7068995

[ref27] KelaiditiE.CesariM.CanevelliM.van KanG. A.OussetP. J.Gillette-GuyonnetS.. (2013). Cognitive frailty: rational and definition from an (I.A.N.A./I.A.G.G.) international consensus group. J. Nutr. Health Aging 17, 726–734. doi: 10.1007/s12603-013-0367-224154642

[ref28] Kojima G. Taniguchi Y. Kitamura A. Fujiwara Y. (2020). Is living alone a risk factor of frailty? A systematic review and meta-analysis. Ageing Res. Rev. 59:101048. doi: 10.1016/j.arr.2020.101048, PMID: 32173535

[ref29] Landi F. onder G. Cesari M. Russo A. Barillaro C. Bernabei R.. (2005). Pain and its relation to depressive symptoms in frail older people living in the community: an observational study. J. Pain Symptom Manag. 29, 255–262. doi: 10.1016/j.jpainsymman.2004.06.016, PMID: 15781176

[ref30] Li M. Q. Huang H. H. Mou X. Jiang G. X. Cheng Q. H. (2018). Cognitive impairment and influencing factors in 70 years old people in Jianghan oil field. J. Am. Geriatr. Soc. 66, S476–S477. doi: 10.3969/j.issn.1009-0126.2017.11.005

[ref31] López-Ortiz S. Caruso G. Emanuele E. Menéndez H. Peñín-Grandes S. Guerrera C. S.. (2024). Digging into the intrinsic capacity concept: can it be applied to Alzheimer's disease? Prog. Neurobiol. 234:102574. doi: 10.1016/j.pneurobio.2024.102574 38266702

[ref32] Malfliet A. Coppieters I. van Wilgen P. Kregel J. de Pauw R. Dolphens M.. (2017). Brain changes associated with cognitive and emotional factors in chronic pain: a systematic review. Eur. J. Pain 21, 769–786. doi: 10.1002/ejp.1003, PMID: 28146315

[ref33] McBeth J. Chiu Y. H. Silman A. J. Ray D. Morriss R. Dickens C.. (2005). Hypothalamic-pituitary-adrenal stress axis function and the relationship with chronic widespread pain and its antecedents. Arthritis Res. Ther. 7, R992–R1000. doi: 10.1186/ar1772, PMID: 16207340 PMC1257426

[ref34] Merchant R. A. Chen M. Z. Tan L. W. L. Lim M. Y. Ho H. K. van Dam R. M. (2017). Singapore healthy older people everyday (HOPE) study: prevalence of frailty and associated factors in older adults. J. Am. Med. Dir. Assoc. 18, 726–728. doi: 10.1016/j.jamda.2017.04.021, PMID: 28623152

[ref35] Montero-Odasso M. M. Barnes B. Speechley M. Muir Hunter S. W. Doherty T. J. Duque G.. (2016). Disentangling cognitive-frailty: results from the gait and brain study. J. Gerontol. A Biol. Sci. Med. Sci. 71, 1476–1482. doi: 10.1093/gerona/glw044 26984391

[ref37] Panza F. Seripa D. Solfrizzi V. Tortelli R. Greco A. Pilotto A.. (2015). Targeting cognitive frailty: clinical and neurobiological roadmap for a single complex phenotype. J. Alzheimers Dis. 47, 793–813. doi: 10.3233/JAD-150358 26401761

[ref38] Panza F. Solfrizzi V. Barulli M. R. Santamato A. Seripa D. Pilotto A.. (2015). Cognitive frailty: a systematic review of epidemiological and neurobiological evidence of an age-related clinical condition. Rejuvenation Res. 18, 389–412. doi: 10.1089/rej.2014.1637, PMID: 25808052

[ref39] Pitcher M. H. Von Korff M. Bushnell M. C. Porter L. (2018). Prevalence and profile of high-impact chronic pain in the United States. J. Pain 20, 146–160. doi: 10.1016/j.jpain.2018.07.006, PMID: 30096445 PMC8822465

[ref40] Robertson D. A. Savva G. M. Kenny R. A. (2013). Frailty and cognitive impairment: a review of the evidence and causal mechanisms. Ageing Res. Rev. 12, 840–851. doi: 10.1016/j.arr.2013.06.004, PMID: 23831959

[ref41] Rong W. Zhang C. Zheng F. Xiao S. Yang Z. Xie W. (2021). Persistent moderate to severe pain and long-term cognitive decline. Eur. J. Pain 25, 2065–2074. doi: 10.1002/ejp.1826, PMID: 34155725

[ref42] RoppoloM.MulassoA.RabagliettiE. (2017). Cognitive frailty in Italian community-dwelling older adults: prevalence rate and its association with disability. Nutr Health Aging 21, 631–636. doi: 10.1007/s12603-017-0766-928537326

[ref43] Searle S. D. Mitnitski A. Gahbauer E. A. Gill T. M. Rockwood K. (2008). A standard procedure for creating a frailty index. BMC Geriatr. 8:24. doi: 10.1186/1471-2318-8-24, PMID: 18826625 PMC2573877

[ref44] Shega J. W. Andrew M. Kotwal A. Lau D. T. Herr K. Ersek M.. (2013). Relationship between persistent pain and 5-year mortality: a population-based prospective cohort study. J. Am. Geriatr. Soc. 61, 2135–2141. doi: 10.1111/jgs.12554, PMID: 24320761 PMC4140782

[ref45] ShimadaH.MakizakoH.LeeS.DoiT.LeeS.TsutsumimotoK.. (2016). Impact of cognitive frailty on daily activities in older persons. J. Nutr. Health Aging 20, 729–735. doi: 10.1007/s12603-016-0685-227499306

[ref46] Smith L. K. He Y. Park J. S. Bieri G. Snethlage C. E. Lin K.. (2015). Beta 2-microglobulin is a systemic pro-aging factor that impairs cognitive function and neurogenesis. Nat. Med. 21, 932–937. doi: 10.1038/nm.3898, PMID: 26147761 PMC4529371

[ref47] Solfrizzi V. Scafato E. Lozupone M. Seripa D. Giannini M. Sardone R.. (2017). Additive role of a potentially reversible cognitive frailty model and inflammatory state on the risk of disability: the Italian longitudinal study on aging. Am. J. Geriatr. Psychiatry 25, 1236–1248. doi: 10.1016/j.jagp.2017.05.018, PMID: 28689645

[ref48] St John P. D. Tyas S. L. Griffith L. E. Menec V. (2017). The cumulative effect of frailty and cognition on mortality - results of a prospective cohort study. Int. Psychogeriatr. 29, 535–543. doi: 10.1017/S1041610216002088, PMID: 27903307

[ref36] Sun H.-L. Bai W. Chen P. Zhang L. Smith R. D. Su Z. (2024). Pain trajectories and their associations with cognition among older adults: a 10-year cohort study from network perspective. Age Ageing 53:afae054. doi: 10.1093/ageing/afae054 38521972 PMC10960922

[ref49] Trevisan C. Veronese N. Maggi S. Baggio G. Toffanello E. D. Zambon S.. (2017). Factors influencing transitions between frailty states in elderly adults: the Progetto Veneto Anziani longitudinal study. J. Am. Geriatr. Soc. 65, 179–184. doi: 10.1111/jgs.14515, PMID: 27861714

[ref50] Varadhan R. Walston J. Cappola A. R. Carlson M. C. Wand G. S. Fried L. P. (2008). Higher levels and blunted diurnal variation of cortisol in frail older women. J Gerontol Med Sci. 63, 190–195. doi: 10.1093/gerona/63.2.190, PMID: 18314456

[ref51] Vos T. Allen C. Arora M. (2016). Global, regional, and national incidence, prevalence, and years lived with disability for 310 diseases and injuries, 1990–2015: a systematic analysis for the global burden of disease study 2015. Lancet 388, 1545–1602. doi: 10.1016/S0140-6736(16)31678-6, PMID: 27733282 PMC5055577

[ref52] Wade K. F. Marshall A. Vanhoutte B. Wu F. C. O’Neill T. W. Lee D. M. (2017). Does pain predict frailty in older men and women? Findings from the English longitudinal study of ageing (ELSA). J. Gerontol. A Biol. Sci. Med. Sci. 72, 403–409. doi: 10.1093/gerona/glw226, PMID: 27836906 PMC5861874

[ref53] Xiong N. Shen J. Wu B. Yan P. P. Shi H. M. Li J.. (2019). Factors influencing cognitive function in patients with atrial fibrillation: a cross-sectional clinical study. J. Int. Med. Res. 47, 6041–6052. doi: 10.1177/0300060519882556, PMID: 31642379 PMC7045671

[ref54] Yan Y. Du Y. Li X. Ping W. Chang Y. (2023). Physical function, ADL, and depressive symptoms in Chinese elderly: evidence from the CHARLS.Front. Public Health 11:1017689. doi: 10.3389/fpubh.2023.1017689, PMID: 36923048 PMC10010774

[ref55] Yang F. Chen Q. W. (2018). Evaluation of frailty and influencing factors in old people in hospital institution: evidence for a phenotype of frailty. Medicine 97:e9634. doi: 10.1097/MD.0000000000009634, PMID: 29504994 PMC5779763

[ref56] Yuan Y. Peng C. Burr J. A. Lapane K. L. (2023). Frailty, cognitive impairment, and depressive symptoms in Chinese older adults: an eight-year multi-trajectory analysis. BMC Geriatr. 23:843. doi: 10.1186/s12877-023-04554-1, PMID: 38087208 PMC10717397

