# Supplementary material for: Association between chronic pain and cognitive frailty among middle-aged and elderly individuals: evidence from the China Health and Retirement Longitudinal Study
Source: Front Aging Neurosci. 2024 Dec 2;16:1491120. doi: 10.3389/fnagi.2024.1491120 (PMC11646885; doi:10.3389/fnagi.2024.1491120)
Supplement: Supplementary file 1 [file Table_1.DOCX]

**Supplemental Table 1. Distribution of frailty index indicators（Referencing literature 37）**

| Description of the items | | | Cut-off value |
| --- | --- | --- | --- |
| Diagnosis ^a^ | Hypertension | | Yes = 1, No = 0 |
|  | Diabetes or high blood sugar | | Yes = 1, No = 0 |
|  | Cancer or malignant tumor | | Yes = 1, No = 0 |
|  | Chronic lung disease | | Yes = 1, No = 0 |
|  | Heart attack, coronary heart disease, angina, congestive heart failure or other heart problems | | Yes = 1, No = 0 |
|  | Stroke | | Yes = 1, No = 0 |
|  | Emotional, nervous, psychiatric problems | | Yes = 1, No = 0 |
|  | Arthritis or rheumatism | | Yes = 1, No = 0 |
|  | Dyslipidemia | | Yes = 1, No = 0 |
|  | Liver disease | | Yes = 1, No = 0 |
|  | Kidney disease | | Yes = 1, No = 0 |
|  | Stomach or other digestive disease | | Yes = 1, No = 0 |
|  | Asthma | | Yes = 1, No = 0 |
| Disability ^b^ | Physical disabilities | | Yes = 1, No = 0 |
|  | Brain damage/mental retardation | | Yes = 1, No = 0 |
|  | Vision problem | | Yes = 1, No = 0 |
|  | Hearing problem | | Yes = 1, No = 0 |
|  | Speech impediment | | Yes = 1, No = 0 |
| Functional limitation ^c^ | Doing household chores | |  |
|  |  | Have difficulty but can still do it | Yes = 1, No = 0 |
|  |  | Have difficulty and need help | Yes = 1, No = 0 |
|  |  | Cannot do it | Yes = 1, No = 0 |
|  | Preparing hot meals | |  |
|  |  | Have difficulty but can still do it | Yes = 1, No = 0 |
|  |  | Have difficulty and need help | Yes = 1, No = 0 |
|  |  | Cannot do it | Yes = 1, No = 0 |
|  | Managing assets | |  |
|  |  | Have difficulty but can still do it | Yes = 1, No = 0 |
|  |  | Have difficulty and need help | Yes = 1, No = 0 |
|  |  | Cannot do it | Yes = 1, No = 0 |
|  | Taking medications | |  |
|  |  | Have difficulty but can still do it | Yes = 1, No = 0 |
|  |  | Have difficulty and need help | Yes = 1, No = 0 |
|  |  | Cannot do it | Yes = 1, No = 0 |
|  | Shopping because of health and memory problems | |  |
|  |  | Have difficulty but can still do it | Yes = 1, No = 0 |
|  |  | Have difficulty and need help | Yes = 1, No = 0 |
|  |  | Cannot do it | Yes = 1, No = 0 |
|  | Running or jogging About 1 kilometer | |  |
|  |  | Have difficulty but can still do it | Yes = 1, No = 0 |
|  |  | Have difficulty and need help | Yes = 1, No = 0 |
|  |  | Cannot do it | Yes = 1, No = 0 |
|  | Getting up from a chair after sitting | |  |
|  |  | Have difficulty but can still do it | Yes = 1, No = 0 |
|  |  | Have difficulty and need help | Yes = 1, No = 0 |
|  |  | Cannot do it | Yes = 1, No = 0 |
|  | Climbing several flights of stairs without rest | |  |
|  |  | Have difficulty but can still do it | Yes = 1, No = 0 |
|  |  | Have difficulty and need help | Yes = 1, No = 0 |
|  |  | Cannot do it | Yes = 1, No = 0 |
|  | Stooping, kneeling, or crouching | |  |
|  |  | Have difficulty but can still do it | Yes = 1, No = 0 |
|  |  | Have difficulty and need help | Yes = 1, No = 0 |
|  |  | Cannot do it | Yes = 1, No = 0 |
|  | Reaching or extending your arms above shoulder | |  |
|  |  | Have difficulty but can still do it | Yes = 1, No = 0 |
|  |  | Have difficulty and need help | Yes = 1, No = 0 |
|  |  | Cannot do it | Yes = 1, No = 0 |
|  | Lifting or carrying weights over 10 jin (about 5 kilograms) | |  |
|  |  | Have difficulty but can still do it | Yes = 1, No = 0 |
|  |  | Have difficulty and need help | Yes = 1, No = 0 |
|  |  | Cannot do it | Yes = 1, No = 0 |
|  | Picking up a small coin from a table | |  |
|  |  | Have difficulty but can still do it | Yes = 1, No = 0 |
|  |  | Have difficulty and need help | Yes = 1, No = 0 |
|  |  | Cannot do it | Yes = 1, No = 0 |

**Notes.**

^a^ Self-reported diagnoses, based on respondents’ answers to the question: “Have you been diagnosed with [conditions] by a doctor.”

^b^ Self-reported disabilities, based on respondents’ answers to the question: “Do you have the following disabilities?”

^c^ Self-reported functional limitations, based on respondents’ answers to the questions “Do you have difficulty with…?”
